# Characteristics of hydrophobicity loss on silicone rubber surface during a dynamic drop test with direct current voltage application

**DOI:** 10.1186/1556-276X-7-4

**Published:** 2012-01-05

**Authors:** Yuya Seo, Norihito Oshikawa, Takuma Miyake, Tatsuya Sakoda, Tomikazu Anjiki

**Affiliations:** 1 Department of Electrical and Electronic Engineering, Graduate School of Engineering, University of Miyazaki, 1-1, Gakuen Kibana-dai Nishi, Miyazaki, 889-2192, Japan; 2 Toshiba Corporation Social Infrastructure Systems Company, Toshiba Hamakawasaki Factory, 2-1, Ukishima, Kawasaki, Kanagawa, 210-0862, Japan

## Abstract

Dynamic drop test for studying the temporal lowering of hydrophobicity on the surface of silicone rubber with direct current voltage application was carried out. In this study, we evaluated the influence of the temporal lowering of hydrophobicity under various conductivities and dropping rates for water droplets. As a result, it was found that the dropping rate and the conductivity of water droplets greatly influenced the hydrophobicity loss time on the surface of silicone rubber.

## Introduction

The development of insulating materials used in electric-powered apparatuses plays an essential role in a stable power supply. Polymer materials, for example, have attracted attention in recent years. The use of polymer materials for housings of insulators and arresters has increased. The widely accepted polymer materials for housings are silicone rubber [SiR] and ethylene vinyl acetate. These polymer materials have some excellent properties such as being lightweight, hydrophobicity, and antiweatherability [1]. Additionally, even if the hydrophobicity initially disappears by external stresses, the hydrophobicity recovers because of the migration of low molecular weight SiR in a short time [2-5].

Incidentally, electric-powered apparatuses are utilized not only by alternating current [AC] voltage applications, but also by direct current [DC] voltage applications. The polymer material is made from organic matters; therefore, the aged deterioration due to electric discharges and acidic products is worrying. Studies on evaluation methods of insulation deterioration of polymer materials are continuously made by the International Council on Large Electric Systems [CIGRE]. A dynamic drop test [DDT] which can easily evaluate the characteristics of temporal lowering of hydrophobicity on the surface of the polymer material was proposed through a continuous research effort [6]. Here, it should be noted that the AC voltage application is a normal test condition for the DDT. The evaluation on the surface with the DC voltage application has almost not been reported.

In this study, we carried out DDT for evaluating the temporal lowering of hydrophobicity on the surface of silicone rubber with DC voltage application. Our results have contributed to the evaluation of polymer materials' reliability with DC voltage application.

## Experimental methods

**Figure 1:**
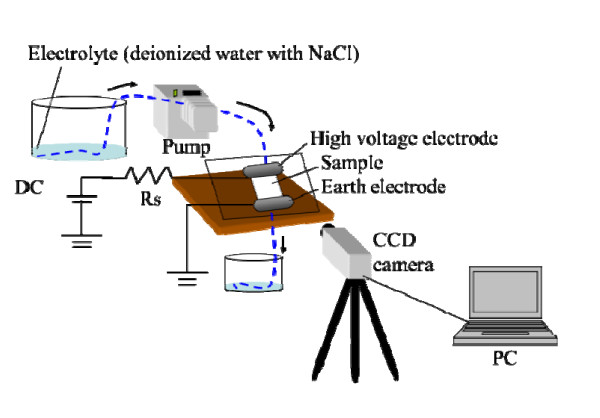
**Experimental setup of DDT with a DC source**.

**Figure 2:**
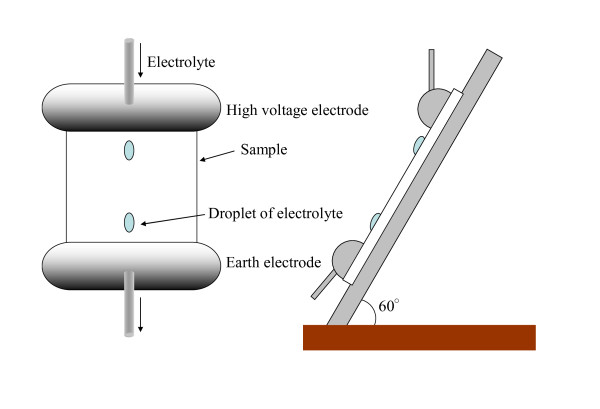
**Configuration of electrodes and a test sample**.

The temporal decrease of hydrophobicity on the surface of the polymer material for the DDT is evaluated by dropping water in small amounts under DC voltage application in DDT. Table 1 shows test conditions of the DDT. Electrolyte was prepared with sodium chloride [NaCl], and conductivity was set at 0.1, 1, 4, 8, 12, and 16 mS/cm. In addition, the dropping rate of electrolyte was set at 12, 24, 36, 48, and 60 drops/min. Simultaneously, DC voltage of DC+3 kV was applied to an upper electrode. Figure 1 shows an experimental setup of the DDT with a DC source. A test sample was arranged between stainless steel electrodes 100 mm in width. A pump (REGLO ISM833, ISMATEC, CSC Central Scientific Comm. Inc., Taito, Tokyo, Japan) drew and dropped the electrolyte continuously to test the sample surface through a small vent of an upper electrode. Droplets on the surface of SiR and discharge phenomena were observed from a charge-coupled device [CCD] camera. Figure 2 shows the configuration of electrodes and a test sample. The test sample used here was SiR, 70 mm in length and 50 mm in width. The distance between electrodes is 60 mm; therefore, the applied electric field was 50 V/mm.

**Table 1:** Test condition of DDT

Item	Specification
Test voltage [kV]	DC+3
Test liquid	Deionized or distilled water with NaCl
Conductivity [mS/cm]	0.1, 1, 4, 8, 12, and 16
Temperature of test liquid [°C]	23 ± 3
Dropping rate [drops/min]	12, 24, 36, 48, and 60

We evaluated the influence of the temporal lowering of hydrophobicity under various conductivities and dropping rates. The changes of hydrophobicity and discharges generated at the surface of the test sample were observed by a CCD camera and shown in Figure 1. The hydrophobicity loss time, when the leakage current of above 2 mA continuously flowed for 4 s from the voltage application, was defined as the same as that proposed by CIGRE.

## Results and discussions

**Figure 3:**
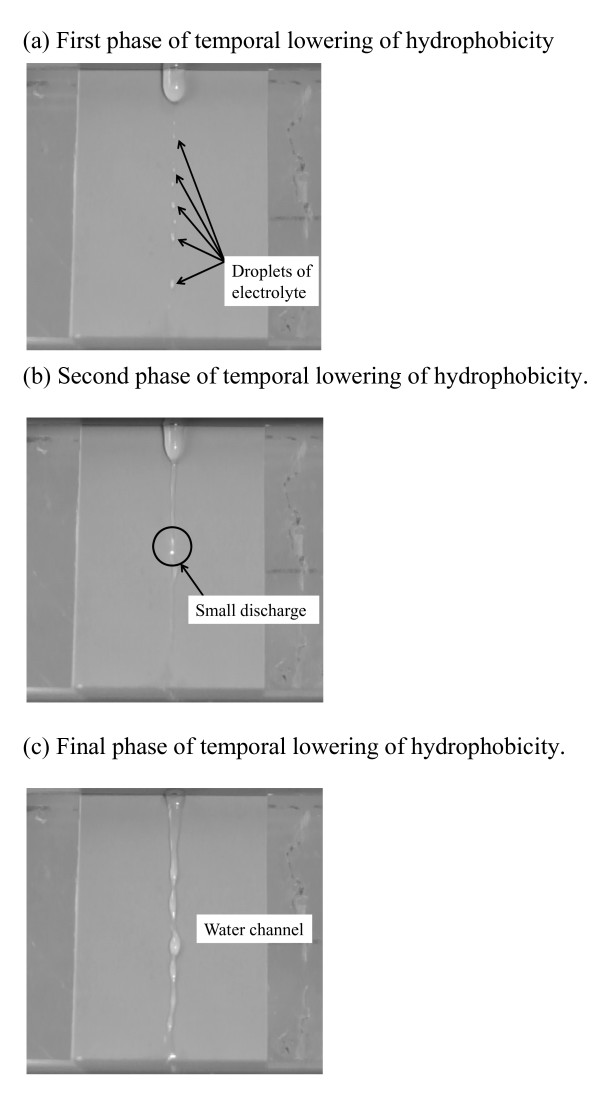
**Typical CCD images of the test sample surface in the temporal lowering of hydrophobicity**. The change of hydrophobicity can be classified into three phases. (**a**) First phase of temporal lowering of hydrophobicity. (**b**) Second phase of temporal lowering of hydrophobicity. (**c**) Final phase of temporal lowering of hydrophobicity.

Figure 3 shows typical CCD images of the temporal lowering of hydrophobicity. As seen from this figure, the change of hydrophobicity can be classified into three phases. With the progress of the lowering of hydrophobicity, small discharges are seen as shown in Figure 3b. In the next stage, as shown in Figure 3c, an obvious water channel is formed.

**Figure 4:**
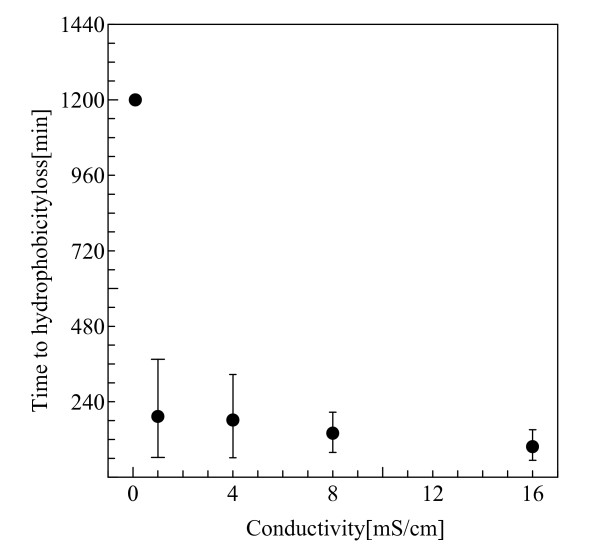
**Hydrophobicity loss time under various conductivities**.

Figure 4 shows the hydrophobicity loss time under various conductivities. As can be seen in this figure, the hydrophobicity loss times for the cases of conductivity above 1 mS/cm were extremely short although that with 0.1 mS/cm was relatively long. Such tendency is probably dependent on NaCl in the electrolyte, i.e., the loss of hydrophobicity resulted from the attachment of NaCl on the surface of the test sample.

**Figure 5:**
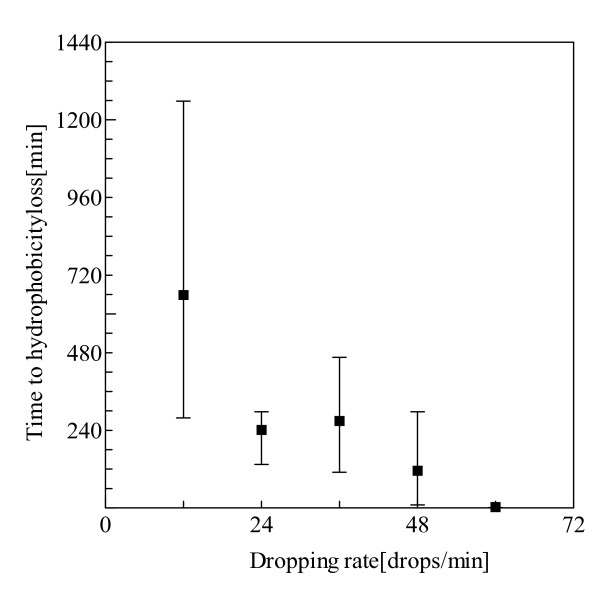
**Hydrophobicity loss time under various dropping rates**.

Figure 5 shows the hydrophobicity loss time under various dropping rates. Additionally, the increase of dropping rate after application of impressed voltage is summarized in Table 2. Needless to say, we first fixed the dropping rate of the electrolyte. However, when we applied electric field to test samples, the number of droplets increased. Notably, in the case of DC voltage application, the increase of droplets was remarkable. In the case of AC voltage application, there is zero-crossing, which is the instantaneous point at which there is no voltage present. However, in the case of DC voltage application, the influence of electrification on the surface of SiR becomes large because there is no zero-crossing.

**Table 2:** The increase of dropping rate after impressed voltage application

Dropping rate before impressed voltage application (drops/min)	Dropping rate after impressed voltage application (drops/min)	
	AC	DC
12	13	16
24	26	29
36	38	43
48	51	56
60	64	70

Thus, due to the influence of the high electric field, the increase of dropping rate was confirmed after impressed voltage was applied. Such increase of droplets is remarkable with the increase of the initial dropping rate of electrolyte. The increase of droplets promoted the lowering of hydrophobicity, and the hydrophobicity loss time became shorter. The surface of the test sample has a greater opportunity to attach NaCl and keeps electrification on the surface of SiR with the increase of the dropping rate.

## Conclusions

We carried out DDT in investigating the temporal lowering phenomena of hydrophobicity of the SiR surface with DC voltage application. Here, the influence of the temporal lowering of hydrophobicity under various conductivities and dropping rates was evaluated. With the progress of the lowering of hydrophobicity, small discharges on the surface of SiR could be seen. Additionally, in the final stage, the hydrophobicity lowered, and an obvious water channel was confirmed. Such hydrophobicity loss was influenced by the conductivity and dropping rate of the electrolyte.

## Competing interests

The authors declare that they have no competing interests.

## Authors' contributions

TS and TA conceived the experiments. YS performed the experiments and analyzed the data together with NO and TM. TS and TA provided valuable advice. TS and TA co-wrote the paper. All authors discussed the results and commented on the manuscript.
